# Health Care Providers’ Perceptions of Quality, Acceptance, and Satisfaction With Telebehavioral Health Services During the COVID-19 Pandemic: Survey-Based Study

**DOI:** 10.2196/23245

**Published:** 2020-12-04

**Authors:** Jesse Wright, Shyam Dewan, Donald Hilty, Naakesh A Dewan

**Affiliations:** 1 BayCare Health System Clearwater, FL United States; 2 Emory University Atlanta, GA United States; 3 University of Southern California Los Angeles, CA United States; 4 Florida Blue Jacksonville, FL United States

**Keywords:** telepsychiatry, COVID-19, telehealth, perception, quality, acceptability, satisfaction, behavior, mental health, health care provider

## Abstract

**Background:**

Due to rapidly increasing rates of COVID-19 across the country, system-wide changes were needed to protect the health and safety of health care providers and consumers alike. Technology-based care has received buy-in from all participants, and the need for technological assistance has been prioritized.

**Objective:**

The objective of this study was to determine the initial perceptions and experiences of interprofessional behavioral health providers about shifting from traditional face-to-face care to virtual technologies (telephonic and televideo) during the COVID-19 pandemic.

**Methods:**

A survey-based study was performed at a large, integrated medical health care system in West-Central Florida that rapidly implemented primary care provision via telephone and televideo as of March 18, 2020. A 23-item anonymous survey based on a 7-point Likert scale was developed to determine health care providers’ perceptions about telephonic and televideo care. The survey took 10 minutes to complete and was administered to 280 professionals between April 27 and May 11, 2020.

**Results:**

In all, 170 respondents completed the survey in entirety, among which 78.8% (134/170) of the respondents were female and primarily aged 36-55 years (89/170, 52.4%). A majority of the respondents were outpatient-based providers (159/170, 93.5%), including psychiatrists, therapists, counselors, and advanced practice nurses. Most of them (144/170, 84.7%) had used televideo for less than 1 year; they felt comfortable and satisfied with either telephonic or televideo mode and that they were able to meet the patients’ needs.

**Conclusions:**

Our survey findings suggest that health care providers valued televideo visits equally or preferred them more than telephonic visits in the domains of quality of care, technology performance, satisfaction of technology, and user acceptance.

## Introduction

Since the first reported case of COVID-19 in the United States in January 2020, social distancing—a misnomer for physical distancing—has been a public health priority [1]. Declarations of public health emergencies and stay-at-home orders have made the use of telephonic and televideo care services a necessity rather than a choice, while also limiting the number of outdoor interactions among the public since March 17, 2020 [2]. During this period, many health care providers worked from home to avoid the risk of exposure to the virus. In general, telehealth options are not always readily available for health care systems due to regulatory, reimbursement, and liability concerns [3]. Nevertheless, owing to patient requests, health care systems have been moving toward the integration of in-person and video services to offer more points-of-service by highly skilled health care providers and to efficiently use available resources [4]. However, changes involving technology are time consuming, compete with other demands, and require considerable investment.

Owing to concerns raised by both health care providers and consumers, and an ongoing state of emergency, the decision to transition to technology-based care has not only pushed but also enabled health care systems to make the change. Buy-in has been received from all participants of care (including health care providers and patients), and technological assistance has been rapidly prioritized. The effort to flatten the curve of COVID-19 spread is an opportunity to “accelerate and bend the curve” of digital health [5]. Fortunately, with the relaxation of HIPAA (Health Insurance Portability and Accountability Act) compliance guidelines, an increasing number of telehealth options are now available and are ready for implementation [6]. Some of the technology options available to the health care system include BlueJeans by Verizon (Verizon Communications), Microsoft Teams (Microsoft Corporation), Skype for Business (Microsoft Corporation), AmWell (American Well Corporation), and Doxy.me (Doxy.me, LLC).

The COVID-19 pandemic has challenged health care systems across the world in ways that both resemble as well as differ from challenges posed by other disasters and mass casualty incidents, including natural phenomena (eg, hurricanes, tornadoes, and wildfires), accidents (eg, plane crashes), or human-made crises (eg, terrorism) [7,8]. These events often cause an acute surge of patients that overwhelms hospital, community, and other resources and personnel. COVID-19 poses infrastructural, communication, and other challenges on a broad scale to responders at various levels—local, state, regional, and federal.

The objective of this study was to determine the initial perceptions of health care providers about the rapid shift in health care delivery—from in-person care to televideo and telephonic care, at a large health care system in Florida that serves acute pediatric, adult, and geriatric populations. The procedures and lessons learned from the implementation of virtual care in this health care system could offer a blueprint for other health care systems. More specifically, this study highlights how health care providers feel about the change from the traditional face-to-face visits to the new patient care approach of telephonic and televideo visits and discusses the advantages and limitations thereof.

## Methods

### Study Overview and Context

This study was conducted at a large, integrated medical health care system in West-Central Florida. The Behavioral Health Division of this system comprised over 750,000 annual outpatient visits and 13,000 inpatient discharges across 5 counties. In anticipation of an emergency order by the Governor of the State, starting March 18, 2020, this health institution decided to proactively shift to a health delivery model offering all care via telephone or televideo. The rationale for this rapid implementation was that the Chief Medical Officer of the Behavioral Health Division felt the need to prioritize patient and staff safety over other factors, such as reimbursement, due to the fear of being overwhelmed by the spread of COVID-19 as in the State of New York [9]. The Behavioral Health Division employs over 1,200 staff, providers, and administrators. These employees had utilized telemedicine to some extent prior to the emergency declaration by the state. However, none of the employed providers had experience using telephonic and televideo solutions in the outpatient setting. Televideo services were rarely used in the inpatient setting, only in cases wherein patient care was absolutely essential.

### Study Design and Outcome

The objective of this survey-based study was to determine health care providers’ initial perceptions of telephonic versus televideo care across 5 domains: (1) quality of care (eg, alliance), (2) technology performance (eg, ability to hear), (3) user experience with technology, (4) satisfaction of technology, and (5) user acceptance. These broad domains are consistent with other behavioral health surveys [10].

An anonymous survey regarding the perceptions of telephonic and televideo care services was sent only to the providers within the Behavioral Health division of this hospital system. Overall, the approach used aligned with the checklist for reporting the results of online surveys [11], in terms of description of the purpose, time taken to complete the survey, voluntary participation, and data protection (ie, anonymous); however, no pretesting was done because of the rapid implementation of the new health care delivery model. Moreover, as this was a quality improvement process, it was approved by the institutional leadership rather than an external or academic institutional review board or human subjects committee. The survey results would offer valuable insights into health care providers’ views on telebehavioral visits for purposes of future enhancements and operations.

### Participants and Procedures

#### Communication With and Inputs From Health Care Providers

Staff and health care providers (survey participants) met with administrators to discuss how to reschedule the upcoming intake of new patients and follow-up appointments, with new patient intake shifted to televideo unless there was no means to do that. Workflows were adjusted based on inputs received from the participants.

#### Technological Adjustments

Coordination was needed among nurses, physicians, therapists, and staff to use information technology. A needs assessment was performed to identify the necessary hardware, software, and other components required to add or supplement existing resources. Various options for televideo (ie, desktop, laptop, cellular phone, and other) were evaluated, but to avoid equipment preparation delays, telephone services were initially chosen. Primary hardware considerations made for health care providers included either a laptop with a built-in web camera or a desktop with an external camera; however, they could also use a mobile smart phone or a tablet device. The software systems selected were Blue Jeans and Microsoft Teams, both of which can be run on any Windows, Android, or Mac device that the provider or patient may have. Technology planning was classified for inpatient and outpatient divisions, as each division required different software and hardware considerations. All outpatient services and programs used the same software and hardware based on its availability at the time.

#### Health Care Providers’ Readiness

Meetings were held with health care providers to assess their needs and provide education to use technology, as well as for basic clinical skills for televideo with a follow-up one-on-one consultation, if requested, particularly for those providers who had not used televideo before. Another challenge was the training and implementation of Microsoft Teams. An operations coordinator provided one-on-one functionality training to each inpatient provider to enable them to perform basic functions using the software. Furthermore, before an upcoming shift, the operations coordinator tested the Microsoft Teams application with each provider to ensure it was downloaded correctly and that audio and video calling features were fully functional.

#### Collaborative Approach

In order to quickly obtain access requirements for part-time health care providers, a collaborative approach was required across multiple departments, including privileging and credentialing, identity access management, data security, and information services.

### Clinical Rollout

As of March 18, 2020, outpatient providers started making telephonic contact with patients, as opposed to in-person visits, to mitigate the risk of COVID-19 spread. Specifically, a provider would reach out to a patient at a scheduled time and conduct an interview from their home (or office while maintaining safe physical distance from other individuals); the patient would also connect from his or her home. Once televideo was known to be a feasible solution, it was chosen to be used across the system for the ambulatory team beginning April 7, 2020, and it was implemented as the primary means to conduct virtual patient visits as of April 22, 2020. The scheduling department sends an email invite to the patient. Then, at a designated time, the patient would need to click on the link in the email to connect with their provider. A similar process was used by therapy providers, whereas field-based providers were required to send a passcode to patients to enable them to join specific meeting rooms at a designated time.

Inpatient providers used 2 software applications that were already in place (ie, Microsoft Teams and Skype for Business) to start providing telehealth services immediately from March 18, 2020. For Microsoft Teams, a clinical operations coordinator created a meeting room using the application so the provider and whoever was using the laptop or device at the inpatient facility could initiate a televideo conference. Once the televideo conference began, the team member in the inpatient unit could take the patient to a private room and help conduct the interview with the provider. In the case of Skype for Business, the provider would have a designated individual at the inpatient facility with whom they would initiate a televideo call. Thereafter, the same process as used for Microsoft Teams was followed with regard to seeing the patients.

### Survey Design and Rollout

The 23-item anonymous survey comprised questions addressing health care providers’ perceptions about telephonic and televideo care. Responses ranged from “Not at all” to “Perfect” on the 1- to 7-point Likert scale, respectively. Participation in the survey was voluntary with no incentives used. In all, 6 of the 23 questions were based on demographics, whereas the remaining 16 questions directly asked respondents the same questions about their perceptions of telephone and televideo care, separately. One of the questions inquired how well the provider saw the patient, which was applicable only to televideo care. The survey took about 10 minutes to complete and was conducted between April 27 and May 11, 2020; that is, it was rolled out 5 days after televideo was chosen as the primary means for care provision in order to understand the providers’ initial perceptions about this mode of virtual care. Our survey was modified based on a survey previously used in a randomized trial [10], but it was not reassessed for validity or internal consistency. Instead of a generalized view on telehealth, our survey was modified to seek opinions to compare the 2 methods used, namely, telephone and televideo. One of the authors is a pioneer in the use of internet-based surveys and evaluating user acceptance and satisfaction of behavioral health technology [12,13].

### Survey Respondents

This closed survey was sent to a total of 280 professionals in the behavioral health division, including psychiatrists, therapists, counselors, and advanced practice nurses (APRNs). The health care providers received the survey via email, with a clickable link directing them to a SurveyMonkey (SVMK Inc.) webpage. Emails were sent to a limited number of inpatient providers who were using either telephonic or televideo solutions and all outpatient providers across the 5 counties serviced by the health care system, including practices in both urban and rural locations. We decided to enroll only a limited number of inpatient providers because not all inpatient providers were using telebehavioral solutions at that time.

We received a total of 209 survey responses, of which 170 were fully completed. Due to the software design, we were not able to account for duplicate entries or questions skipped by the respondents. However, at the time of distribution of the survey link, participants were notified to complete the survey only once and in its entirety.

### Statistical Analysis

To determine initial differences of perceptions among users regarding the use of telephone and televideo services, analyses were performed using paired *t* tests.

## Results

Responses on all questions were collected at a completion rate of 60.7% (170/280). Surveys that were not fully completed were not included in this analysis. In all, 78.8% (134/170) of the respondents were female, stratified into the following age ranges: 20-35 years (48/170, 28.2%), 36-55 years (89/170, 52.4%), and ≥56 years (33/170, 19.4%). With regard to the location of care, 93.5% (159/170) of respondents were outpatient providers and 6.5% (11/170) were inpatient providers. The professional backgrounds of the respondents also varied considerably, as follows: physicians (22/170, 12.9%), licensed MH therapists (54/170, 31.8%), social workers (22/170, 12.9%), APRNs (9/170, 5.3%), Bachelor’s degree holders (27/170, 15.9%), unlicensed Master’s degree holders (31/170, 18.2%), and psychiatric support (5/170, 2.9%). The majority of respondents had less than 1 year of experience with telephone (138/170, 81.2%) and televideo care (144/170, 84.7%).

The survey results suggest that health care providers valued televideo mode equally or preferred it more than telephonic mode in the domains of quality of care, technology performance, satisfaction of technology, and user acceptance. Table 1 shows differences in the scores for these domains in terms of developing patient-clinician alliance, meeting the patients’ needs, and evaluating the experience relative to face-to-face care. The following 2 aspects were of particular importance: (1) the ability to provide care as well as in a face-to-face visit and (2) how well the providers met the patients’ needs via telephonic and televideo visits, with corresponding scores of 3.92 versus 4.48 (*t*=3.51, *P*<.001) and 4.65 versus 5.12 (*t*=3.41, *P*<.001), for telephonic and televideo visits, respectively. This finding indicates that televideo was preferred for both these aspects. The biggest difference between telephonic and televideo perceptions was observed in response to the question about the patient-clinician alliance (3.98 versus 4.89, *t*=7.17, *P*<.001), again indicating a preference for televideo.

**Table 1:** Survey responses from health care providers.

Domain and survey question		Score				
			Telephonic visit, mean score	Televideo visit, mean score	*P* value	*t* value
**Quality of care**						
	How well did you develop the patient-clinician alliance?		3.98	4.89	*<*.001	7.17
	How well did you meet the patient’s needs?		4.65	5.12	<.001	3.41
	Was the care as good as a face-to-face?		3.92	4.48	<.001	3.51
	How freely were you able to talk about patient issues?		4.70	4.88	.20	1.29
**Technology performance**						
	How well were you able to hear the patient?		5.03	4.85	.14	1.48
**Satisfaction of technology**						
	How satisfied were you with the experience?		4.35	4.86	.0033	2.96
**User acceptance**						
	How would you rate your sophistication?		5.02	5.26	.094	1.68
	How comfortable are you using this technology?		5.36	5.62	.11	1.62

## Discussion

We believe this is the first study to survey health care providers across disciplines and settings regarding their initial perceptions of telephonic and televideo visits during a rapid transition from face-to-face visits during the COVID-19 pandemic. Preference for televideo visits over telephonic visits was slight but significant. As health care systems, providers, technology companies, and payers struggle with how, when, and to what extent should technology be utilized [14], the findings of this survey-based study suggest that providers are ready and capable of using various means for interacting with patients. The approach used to introduce technologies is consistent with other health care systems, which have emphasized the need for obtaining inputs from staff and clinicians, notifying patients, and committing to a plan of action and trial periods in order to improve outcomes [15].

However, our study has some limitations. First, the questionnaire was not tested for validity and reliability. Second, it was a brief survey, comprising only a few questions for each domain. Third, although questions for each domain were adapted from existing questionnaires that are probably reliable and valid, the survey could have been retested as well as checked for internal consistency. Fourth, self-reporting methods allow scope for improvement, without further validation. Fifth, ideally, we would compare results from in-person, telephonic, and televideo visit groups. While our methods could have been improved, we think the findings from this study provide valuable insights in terms of ongoing operations and strategic planning. Future efforts are needed to validate this survey and provide a metric for further evaluations of health care providers’ perceptions of telephonic and televideo visits. Finally, the results may not be generalizable to other health care systems, in terms of the providers surveyed, the dimensions of the health care system, and other aspects. Therefore, further studies should evaluate patient satisfaction and acceptance, long-term quality, and ramifications of therapeutic alliances. These studies should also explore concerns among the health care providers, such as anxiety about care via televideo, telephone, and other virtual technologies, as well as other dimensions of care provision during the COVID-19 pandemic.

The US health care system will need to make considerable efforts to adapt to new, post-pandemic norms, and individual health care systems will need to evaluate and review the role of technology in providing patient care. As a result, institutional leadership is needed to support technological interventions so that clinical, technological, and administrative operations can ensure the wellbeing of providers and the health of patients as well as the community at large [16]. In addition, to ensure quality of care, health care systems could benefit from more directly assessing providers’ skills, prioritizing specific ones, and aligning the implementation of technology training with competencies, such as those for video [17,18], social media [19], mobile health [20], and asynchronous health care [21]. Telehealth may also afford opportunities to reach new populations, help underserved ones, and build relationships with community partners [21].

## Abbreviations

APRNs: advanced practice nurses
HIPAA: Health Insurance Portability and Accountability Act
